# Comparative Evaluation of Polymer Screening and Oil Displacement Performance in Class III Reservoirs of the Daqing Oilfield

**DOI:** 10.3390/polym18020147

**Published:** 2026-01-06

**Authors:** Ming Yu, Yunwei He, Xin Jin, Tong Pei, Jinyun Wei, Fushan Li, Shuaishuai Zhao, Yanfu Pi

**Affiliations:** 1 Geological Research Institute of the Sixth Oil Production Plant, Daqing Oilfield Company Limited, Daqing 163114, China; 2 Key Laboratory of Enhanced Oil and Gas Recovery of Ministry of Education, Northeast Petroleum University, Daqing 163318, China

**Keywords:** polymer flooding, polymer screening, enhanced oil recovery, Class III reservoir

## Abstract

Class III reservoirs in the Daqing Oilfield are characterized by low permeability and strong heterogeneity, posing significant challenges to enhanced oil recovery (EOR). To improve the recovery efficiency of these reservoirs, the viscosifying ability, stability, shear resistance, and profile-control performance of fifteen polymer solutions were experimentally evaluated, and the two most compatible formulations were selected for the Daqing Class III reservoirs. Subsequently, a three-dimensional physical model equipped with real-time saturation monitoring was employed to compare the EOR performance of the selected polymers. The results indicate that a 1500 mg L^−1^ polymer solution with a molecular weight (Mw) of 16 × 10^6^ Da and a 1200 mg L^−1^ polymer solution with an Mw of 19 × 10^6^ Da exhibit the best compatibility with the target formation. After injecting the 1500 mg L^−1^ (Mw = 16 × 10^6^ Da) polymer solution, the ultimate recovery reached 53.38%, with displacement efficiencies of 64.34% and 58.16% and sweep efficiencies of 92.26% and 80.35% in the high- and low-permeability layers, respectively. Injection of the 1200 mg L^−1^ (Mw = 19 × 10^6^ Da) polymer solution yielded an overall recovery of 47.71%, corresponding to displacement efficiencies of 60.34% and 54.16% and sweep efficiencies of 88.52% and 76.38%. Consequently, the 1500 mg L^−1^ (Mw = 16 × 10^6^ Da) polymer solution delivers the highest recovery increment in Class III reservoirs. These findings provide valuable guidance for the efficient polymer-flooding development of Class III reservoirs in Daqing and analogous formations worldwide.

## 1. Introduction

In recent years, the total consumption of fossil fuels has continued to grow, still accounting for a large proportion of the energy structure [1,2,3]. With long-term production and extraction, some oil fields have entered the high-water or even ultra-high-water stage. After entering the high-water stage, conventional water injection techniques can no longer effectively displace crude oil, often resulting in ineffective water injection cycles that consume substantial resources and costs. As a result, many oil fields have turned to enhanced oil recovery (EOR) technologies, with polymer flooding being the most widely used method. Polymer flooding is mainly applied during the tertiary recovery stage, where polymers increase the viscosity of the displacement fluid and improve the oil–water mobility ratio, effectively expanding the sweep volume and enhancing the recovery rate [4,5,6,7].

Numerous experiments and field practices have demonstrated that polymer flooding performs well in reservoirs with moderate to high permeability and relatively weak heterogeneity. However, in low-permeability and strongly heterogeneous reservoirs, the applicability and recovery mechanisms of polymer flooding remain challenging and not well understood. Daqing Oilfield classifies reservoirs into Class I (400–500 × 10^−3^ μm^2^), Class II (200–300 × 10^−3^ μm^2^), and Class III, with Class III reservoirs primarily referring to oil layers with a single effective thickness of 0.5–1.0 m and an effective permeability of less than 200 × 10^−3^ μm^2^. Currently, Class III reservoirs in the Lama Dian Oilfield have become the main tertiary recovery target after Class I and II reservoirs, with significant potential for EOR. Compared to Class I and II reservoirs, Class III reservoirs are characterized by many thin layers, low permeability, poor connectivity, complex phase changes, and strong heterogeneity [8,9,10,11,12], which causes the fluid to preferentially bypass high-permeability layers during conventional polymer flooding, with insufficient displacement in low-permeability zones, limiting the overall sweep efficiency and recovery rate. Therefore, there is an urgent need to carry out targeted polymer system screening and performance evaluation for Class III reservoirs.

Many scholars have investigated factors affecting polymer flooding efficiency. Song et al. [13] compared various polymers used in China, noting that stability, injectivity, and shear resistance are the main factors influencing polymer flooding efficiency. Yang et al. [14] studied the application of zwitterionic polymers/organic chromium gels in high-salinity reservoirs, finding that stability significantly affects polymer EOR performance. Jin et al. [15] studied the improvement in water absorption profiles during polymer injection through double-tube core flooding experiments, revealing that polymer injection effectively improves the water absorption profile.

Previous studies mainly focus on polymer rheological properties [16,17], thermal stability [18], chemical stability [19,20], injectivity [21,22], adsorption, and degradation behavior [23,24,25]. These studies often use relatively homogeneous core conditions for evaluation [26,27]. Some studies have introduced heterogeneous cores or physical models, but most focus on a single or few performance indicators, making it difficult to reflect the comprehensive adaptability of polymer systems in strongly heterogeneous reservoirs. Particularly, quantitative comparison of polymer flooding performance between high- and low-permeability layers under Class III reservoir conditions remains limited, which restricts the effective translation of experimental results into engineering applications.

Based on the above background, this study aims to establish a polymer flooding evaluation and comparison framework for low-permeability, strongly heterogeneous Class III reservoirs in the Daqing Oilfield. Partially hydrolyzed polyacrylamide (HPAM) is selected as the polymer system, and 15 polymer solutions with different molecular weights and concentrations are systematically evaluated using multiple performance indicators, including viscosity enhancement, stability, shear resistance, injectivity, and profile control capacity. Two polymer solutions exhibiting the best compatibility with Class III reservoirs are identified, and a three-dimensional physical model combined with oil saturation monitoring is employed to quantitatively compare the displacement efficiency and sweep characteristics of these polymer systems in high- and low-permeability layers.

The main innovations and contributions of this study are as follows:

(1) A multi-criteria polymer screening and evaluation approach tailored to Class III reservoir development conditions is proposed and applied, overcoming the limitations of single-parameter evaluations;
(2) A three-dimensional heterogeneous physical model is integrated with oil saturation monitoring techniques to enable a quantitative comparison of polymer flooding performance in high- and low-permeability layers;
(3) The matching relationship between polymer molecular weight and concentration in strongly heterogeneous Class III reservoirs, and its impact on sweep efficiency and ultimate oil recovery, is experimentally elucidated.

**Figure 1:**
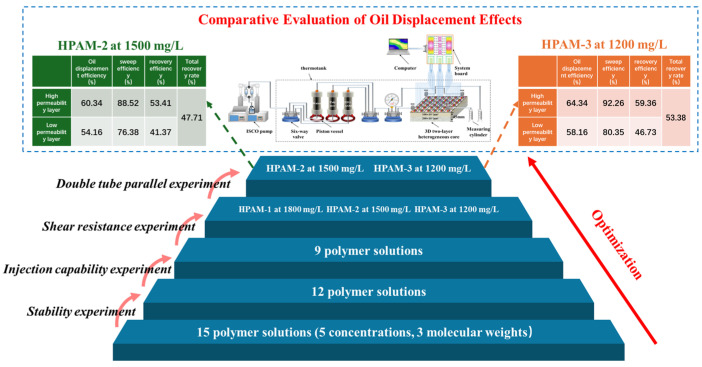
Research workflow for polymer screening and evaluation in Class III reservoirs of the Daqing Oilfield.

The research framework of this study is shown in Figure 1. First, the viscosity and stability of 15 polymer solutions were tested. Based on injectivity experiments, 9 polymer solutions were selected for shear resistance tests, and 3 polymers with high shear resistance were further tested in the double-tube core flooding experiment. Finally, 2 polymer solutions with strong profile control were selected. The displacement efficiency and sweep coefficient of these two polymer solutions were compared using the three-dimensional physical model, combined with oil saturation monitoring equipment. The results of this study provide experimental evidence and technical guidance for polymer flooding scheme design and parameter optimization for Class III reservoirs in Daqing Oilfield and similar reservoirs in other oil fields.

## 2. Materials and Methods

### 2.1. Experimental Materials

Partially hydrolyzed polyacrylamide (HPAM) with molecular weights of 1200 × 10^4^ (HPAM-1), 1600 × 10^4^ (HPAM-2), and 1900 × 10^4^ (HPAM-3), a hydrolysis degree of 23–27%, and a solid content greater than 88%, was supplied by Daqing Refining & Petrochemical Company, PetroChina(Daqing, China). The fifteen polymer solutions used in the experiments were systematically designed by combining different molecular weight grades (1200 × 10^4^, 1600 × 10^4^, and 1900 × 10^4^) with solution concentrations of 800, 1000, 1200, 1500, and 1800 mg/L. Freshwater and wastewater were provided by the Sixth Oil Production Plant of the Daqing Oilfield (Daqing, China). The water used for core saturation was simulated formation water with a salinity of 6778 mg/L, and its chemical composition is listed in Table 1.

Polymer solutions were prepared using a clean-water-diluted wastewater method. The stock polymer solution (5000 mg/L) was prepared with clean water, and the polymer stock solution was then diluted to the required concentrations using wastewater provided by the site. The crude oil used in the experiments was dehydrated and degassed crude oil from the Sixth Oil Production Plant of Daqing Oilfield (Daqing, China). The simulated oil was prepared by mixing the dehydrated and degassed crude oil with aviation kerosene, achieving a dynamic viscosity of 9.8 mPa·s at 45 °C.

According to the characteristics of the Class III reservoir polymer flooding experimental block, artificial core models with permeabilities of 100 × 10^−3^ μm^2^ and 200 × 10^−3^ μm^2^ were fabricated to simulate the properties of Class III reservoirs. The standard cylindrical cores (100 mm × φ25 mm) used in the injectivity experiments all had a permeability of 100 × 10^−3^ μm^2^. Parameters for the one-dimensional homogeneous core model used in the shear resistance experiments are provided in Table 2. The parameters for the dual-tube parallel model are shown in Table 3. The parameters for the three-dimensional saturation monitoring well network model are shown in Table 4, with 72 pairs of electrodes arranged on the model to allow real-time monitoring of the oil saturation field using the oil saturation monitoring device.

### 2.2. Experimental Methods

#### 2.2.1. Viscosity Increasing Experiment

The polymer stock solution with a concentration of 5000 mg/L was prepared using clean water, and then diluted with wastewater provided by the site to prepare polymer solutions with concentrations of 800 mg/L, 1000 mg/L, 1200 mg/L, 1500 mg/L, and 1800 mg/L. The 15 polymer solutions were allowed to mature for 2 h. The viscosity of the diluted solutions was measured using a DV-II Viscometer at a temperature of 45 °C. To reduce experimental uncertainty, each viscosity measurement was performed in triplicate, and the average value was recorded.

#### 2.2.2. Stability Experiment

The polymer stock solution with a concentration of 5000 mg/L was prepared using clean water, and then diluted with wastewater to obtain five different concentrations as described in the previous section. The diluted solutions were transferred into ampoules, degassed using an iHDAS-II intelligent high-efficiency deoxygenation system, sealed, and then placed in a 45 °C thermostatic oven for aging for 90 days. Polymer viscosity was measured on days 1, 5, 10, 15, 20, 30, 40, 50, 60, 70, 80, and 90. To reduce measurement uncertainty, each viscosity value was measured in triplicate, and the average value was recorded.

#### 2.2.3. Injection Capability Experiment

Core flow experiments were conducted using standard cylindrical cores to evaluate the injectivity of different polymer solutions based on injection pressure. The experimental procedure is as follows: (1) Polymer solutions were filtered using a 3.0 μm microporous filter membrane to prevent any insoluble matter in the solution from causing blockage on the rock surface; (2) The standard 100 × 10^−3^ μm^2^ cylindrical core was vacuum-saturated with water, and the porosity was calculated; (3) Polymer solutions were injected into the core at a volume of 10 PV, with each injection of 0.5 PV recorded at the injection pressure at the injection end.

#### 2.2.4. Shear Resistance Experiment

**Figure 2:**
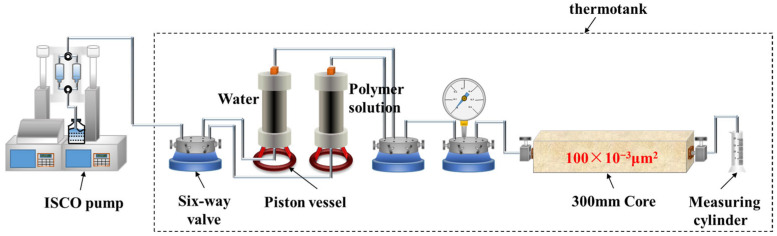
Schematic diagram of the shear-resistance core-flood apparatus.

Artificial homogeneous cores were used to simulate the actual reservoir and conduct core flow experiments to model the shear effect of the porous medium on polymer solutions. The experimental setup and flow diagram are shown in Figure 2. The experimental procedure is as follows: (1) The one-dimensional homogeneous core model was vacuum-saturated with simulated formation water, and the porosity of the core was calculated; (2) At 45 °C, the injection rate was set to 0.3 mL/min (simulating the field injection rate of 1 m/d), and polymer solutions were injected, with 0.2 PV injected per run. Polymer viscosity at the outlet was measured after each injection, and the process was stopped after 2 PV; (3) Another core with the same batch and permeability was used, and step (2) was repeated to test other polymer solutions.

#### 2.2.5. Double Tube Parallel Experiment

**Figure 3:**
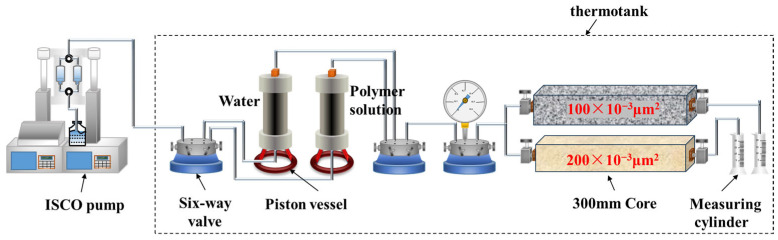
Schematic diagram of the dual-core parallel-flood apparatus.

A dual-tube parallel core model was used to simulate the heterogeneity of the reservoir, with the experimental setup shown in Figure 3. The objective was to examine the splitting ratio in high- and low-permeability cores when using different polymer solutions for enhanced oil recovery and to evaluate the compatibility of the polymer solutions with heterogeneous reservoirs. The experimental procedure is as follows: (1) The core model was vacuumed for 24 h and then saturated with formation water. The pore volume and permeability were measured; (2) The core was saturated with simulated oil at a flow rate of 0.3 mL/min, and the oil saturation was calculated. The core was then aged in a 45 °C thermostatic oven for 7 days; (3) The ISCO pump flow rate was set to 0.3 mL/min, and a waterflooding experiment was conducted until the water cut reached 98%. After injecting 1 PV of polymer solution, the subsequent waterflooding was continued until the water cut reached 98% again. Parameters such as pressure, effluent volume, and oil production were measured throughout the experiment.

#### 2.2.6. Three Dimensional Saturation Monitoring Well Network Model Oil Displacement Experiment

**Figure 4:**
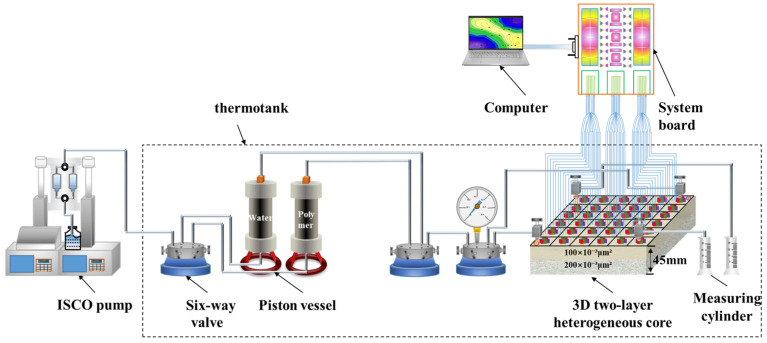
Schematic diagram of the 3D saturation-monitoring well-pattern displacement apparatus.

**Figure 5:**
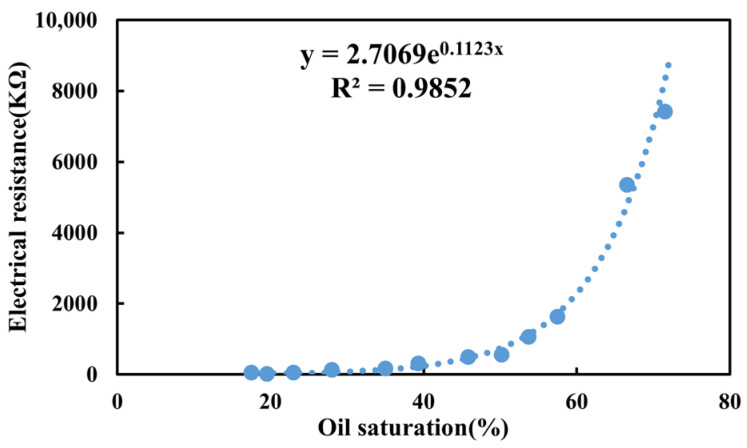
The relationship between resistivity and oil saturation.

**Figure 6:**
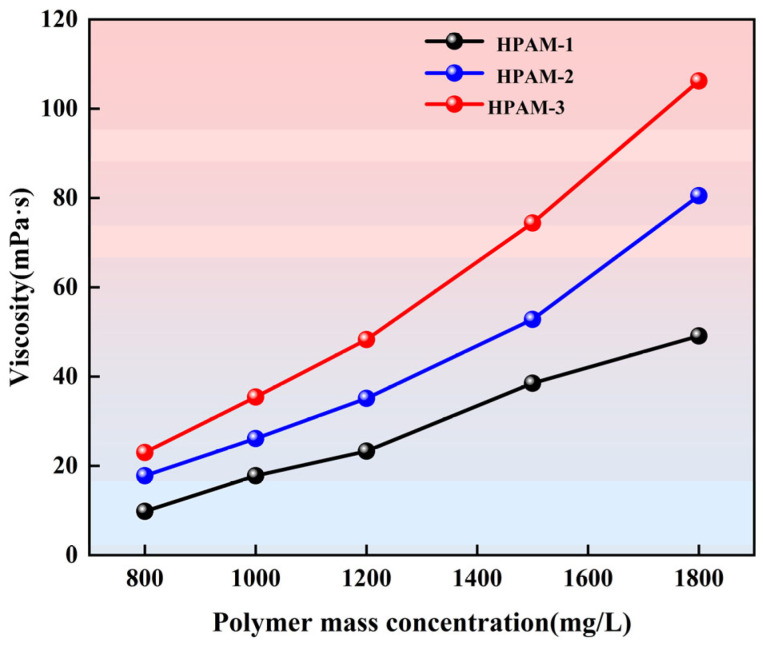
Viscosity–concentration curves of the three HPAM solutions.

Based on actual field conditions, a three-dimensional saturation monitoring well network model was used to conduct polymer flooding experiments, aiming to investigate the macroscopic displacement efficiency and microscopic recovery extent when different polymer solutions are used. The experimental setup and connection diagram are shown in Figure 4. The experimental procedure is as follows: (1) The three-dimensional saturation monitoring well network model was vacuum-saturated with water and then with oil, and aged for 7 days; (2) At 45 °C, the model was injected with formation wastewater at a flow rate of 0.75 mL/min in a one-injection-one-production mode until the water cut reached 98%; (3) The flow rate was kept constant, and 1 PV of polymer solution was injected into the model. Waterflooding was then continued until the water cut reached 98%, and the experiment was completed; (4) During the experiment, parameters such as pressure, effluent volume, oil production, and water production were recorded, and recovery factors and water cut were calculated at each stage; (5) A synthetic core, consistent with the materials used in the three-dimensional physical model, was selected, and its resistance was measured under different known oil saturation conditions. A resistivity vs. oil saturation calibration curve was then established, as shown in Figure 5; (6) The saturation monitoring system was used to record the resistivity of the different permeability layers at each stage of the displacement process. The oil saturation distribution was then calculated based on the resistivity and oil saturation calibration curve, and the saturation field distribution map was plotted.

In order to quantitatively compare the displacement performance of different polymer solutions, the oil displacement efficiency and the sweep coefficient were further calculated based on the saturation maps obtained from the resistivity monitoring system.

Oil displacement efficiency (*E_D_*) is defined as the fraction of movable oil removed from the swept volume:

(1) $$E_{D}=\frac{S_{oi}-S_{or}}{S_{oi}}\times 100\%$$

where *S_oi_* is the initial oil saturation in the considered region (or layer), and *S_or_* is the residual oil saturation after polymer flooding (or after a given stage).

Sweep efficiency (*E_V_*) is defined as the fraction of pore volume contacted by the injected fluid:

(2) $$E_{V}=\frac{V_{s}}{V_{p}}\times 100\%$$

where *V_s_* is the swept pore volume, and *V_p_* is the total pore volume of the considered region (or layer).

## 3. Results and Discussion

### 3.1. Viscosity Increasing Experiment

Polymers with superior viscosifying capability effectively increase the viscosity of the aqueous phase, thereby reducing the water-to-oil mobility ratio and enhancing volumetric sweep efficiency, which is a key displacement mechanism in polymer flooding. The viscosity–concentration relationships of the three polymer solutions at identical concentrations are illustrated in Figure 6.

As shown in Figure 6, the viscosity of all three polymer solutions increases with increasing concentration. At the same concentration, HPAM-3 consistently exhibits a higher viscosity than the other two polymers, and this difference becomes more pronounced when the concentration exceeds 1200 mg/L. As the concentration increases from 800 to 1800 mg/L, the viscosity of HPAM-1 increases from 9.8 to 49.1 mPa·s, that of HPAM-2 from 17.8 to 80.5 mPa·s, and that of HPAM-3 from 23.0 to 106.2 mPa·s. Overall, HPAM-3 shows the highest viscosity across the entire concentration range.

### 3.2. Stability Experiment

**Figure 7:**
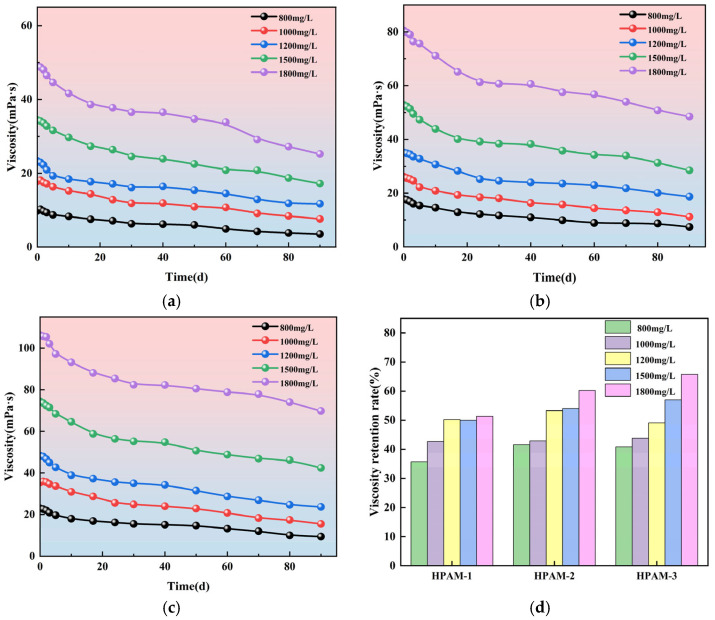
Evolution of polymer-solution viscosity during aging: (**a**) HPAM-1; (**b**) HPAM-2; (**c**) HPAM-3; (**d**) comparison among the three polymers.

The purpose of this aging experiment is not to replicate all the degradation processes that occur in the actual downhole environment, but to compare and evaluate the basic thermal and chemical stability of different polymer systems under controlled conditions. Static thermal aging experiments allow the effect of temperature on polymer molecular structure stability to be isolated, excluding the influence of shear forces and pore structure. As a result, such experiments are commonly used as a fundamental evaluation method during the polymer screening stage. Under identical experimental conditions, the viscosity vs. aging time curves for the 15 polymer solutions are shown in Figure 7.

As shown in Figure 7a–c, the viscosity of the polymer solutions increases with concentration, and the viscosity of all three polymers decreases over the aging period. From panel d, it can be seen that after 90 days of aging, the viscosity retention rate of HPAM-1 polymer is between 35.71% and 51.32%; for HPAM-2, it is between 41.57% and 60.25%; and for HPAM-3, it is between 40.87% and 65.73%. At the same aging time, higher molecular weight and higher concentration polymers exhibit better viscosity retention. This is because in high molecular weight polymer solutions, the interactions between polymer chains are stronger, and the entanglements and hydrogen bonds between chains help form a network structure that enhances the thermodynamic stability of the polymer. The viscosity retention rate of the 800 mg/L polymer solutions for all three types is the lowest and will not be tested in subsequent experiments.

### 3.3. Injection Capability Experiment

**Figure 8:**
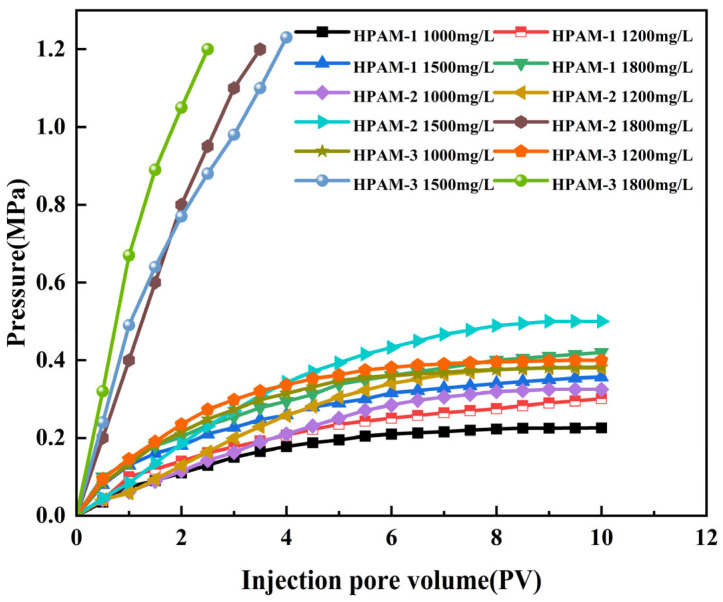
Injection-pressure versus injected-volume curves for the twelve polymer solutions.

Polymer transport through porous media is governed by the relative sizes of the polymer molecules, the pore structure of the medium, and the interactions between polymer chains. When the molecular weight is excessively high, pore plugging may occur, causing the injection pressure to rise continuously with increasing pore volumes injected (PV). Conversely, if the pressure increases rapidly at the beginning and then gradually plateaus to a stable value, no plugging is indicated. Based on this principle, the pressure–PV relationships of twelve polymer solutions were evaluated using standard cylindrical cores; the results are presented in Figure 8.

The criteria used to distinguish polymer injectivity from flow restriction are defined as follows: when, at an early injection stage (PV < 3), the differential pressure rises rapidly, exceeds approximately 0.8 MPa, and exhibits a pronounced nonlinear escalation, the polymer system is considered to experience severe flow restriction. In contrast, when the differential pressure increases gradually with injected pore volume and approaches a stable trend, with the maximum differential pressure remaining below 0.5 MPa, the polymer system is regarded as possessing sustainable injectivity.

As shown in Figure 8, during injection of HPAM-2 at 1800 mg/L (80.5 mPa·s), HPAM-3 at 1500 mg/L (74.4 mPa·s), and HPAM-3 at 1800 mg/L (106.2 mPa·s), the injection-end pressure increased continuously and sharply within a short injection volume, indicating injection blockage. These results demonstrate that the three polymer solutions are difficult to inject under Class III reservoir conditions. In contrast, the remaining polymer solutions exhibit lower injection pressures and a more gradual pressure increase, allowing smooth and sustained injection. Consequently, the three polymer solutions identified as injection-blocked were excluded from subsequent experiments.

### 3.4. Shear Resistance Experiment

During solution preparation and injection, polymer solutions are subjected to varying degrees of mechanical shear, which leads to viscosity degradation and can substantially affect polymer flooding performance. In this study, shear resistance is characterized by changes in the viscosity of the produced fluid, with emphasis placed on its practical implications for sweep volume and enhanced oil recovery.

**Figure 9:**
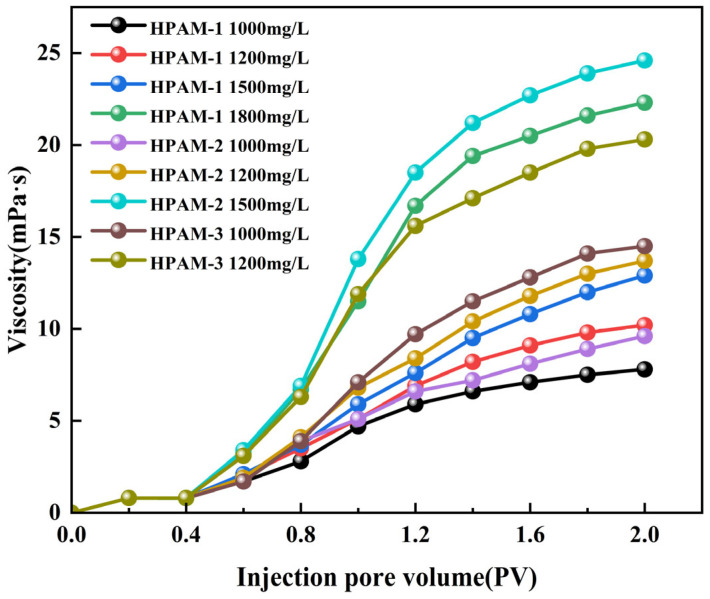
Effluent viscosity versus injected pore-volume multiple for the polymer solutions.

Polymer solutions were injected into a one-dimensional homogeneous core model to simulate viscosity changes induced by shear within porous media. The experimental results are presented in Figure 9.

As shown in Figure 9, the outlet viscosity increases with increasing injected pore volume for all polymer solutions. When the initial viscosity of the polymer solution is lower than 35 mPa·s, the viscosity after shear through the porous medium decreases to below 15 mPa·s. In contrast, when the initial viscosity is approximately 50 mPa·s, the post-shear viscosity remains higher than 20 mPa·s.

The reduction in viscosity after shear through porous media is mainly attributed to several mechanisms. Under strong shear stress within the porous medium, the originally long, entangled polymer molecular chains undergo scission, leading to a sharp decrease in intermolecular interactions. In addition, shear disrupts the polymer molecular coils, resulting in a reduction in the hydrodynamic radius of the polymer chains. Partial breakage of polymer side chains further contributes to the decrease in apparent solution viscosity.

Based on the shear resistance experiments, HPAM-1 at 1800 mg/L, HPAM-2 at 1500 mg/L, and HPAM-3 at 1200 mg/L retain relatively higher effective in-reservoir viscosities after shear, indicating superior shear resistance. Therefore, these three polymer solutions were selected for subsequent experiments.

### 3.5. Double Tube Parallel Experiment

**Figure 10:**
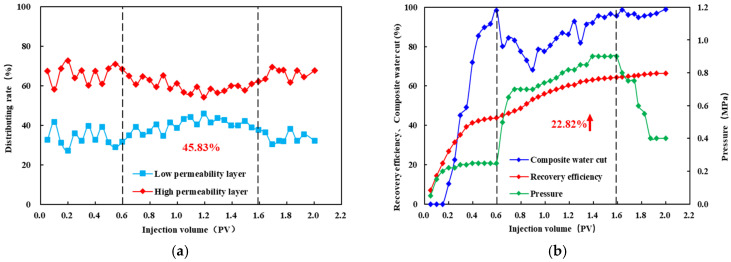
Diverting-flow and production performance for HPAM-1 at 1800 mg L^−1^: (**a**) fractional flow to the low-permeability layer versus injected volume; (**b**) cumulative recovery curve. The arrow highlights the increase in recovery efficiency (22.82%) during the polymer flooding stage.

**Figure 11:**
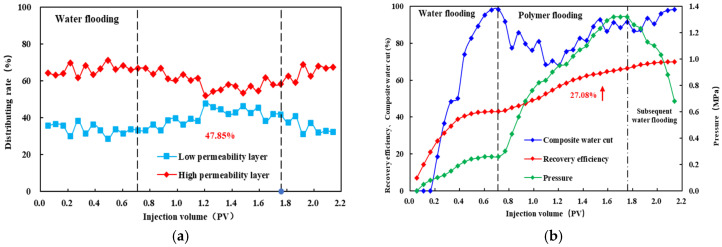
Diverting-flow and production performance for HPAM-2 at 1500 mg L^−1^: (**a**) fractional flow to the low-permeability layer versus injected volume; (**b**) cumulative recovery curve. The arrow highlights the increase in recovery efficiency (27.08%) during the polymer flooding stage.

**Figure 12:**
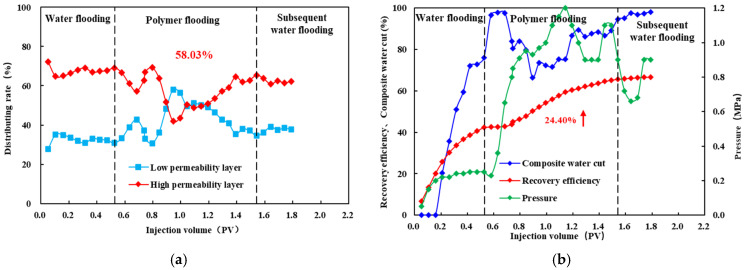
Diverting-flow and production performance for HPAM-3 at 1200 mg L^−1^: (**a**) fractional flow to the low-permeability layer versus injected volume; (**b**) cumulative recovery curve. The arrow highlights the increase in recovery efficiency (24.40%) during the polymer flooding stage.

As shown in Figure 10, Figure 11 and Figure 12, during the polymer flooding stage, the flow diversion ratio of the low-permeability layer initially increases and then decreases. This behavior can be attributed to the evolution of polymer distribution during injection. At the early stage of polymer injection, the polymer preferentially enters the high-permeability layer and partially restricts flow within this layer, thereby reducing its fluid intake and improving diversion toward the low-permeability layer. With continued injection, polymer retention occurs in the high-permeability layer, which subsequently promotes the redistribution of the injected polymer solution toward the low-permeability layer, leading to a deterioration of the diversion effect at later stages.

After injecting HPAM-1 at a concentration of 1800 mg/L, the maximum diversion ratio of the low-permeability layer reached 45.83%, accompanied by a recovery factor increase of 22.82%. For HPAM-2 at 1500 mg/L, the maximum low-permeability-layer diversion ratio was 47.58%, with a recovery improvement of 27.08%. In the case of HPAM-3 at 1200 mg/L, the low-permeability layer achieved the highest maximum diversion ratio of 58.03%, corresponding to a recovery increase of 24.40%.

In summary, significant differences are observed in the profile control performance among the polymer systems. The oil production performance during the polymer flooding stage indicates that HPAM-2 at 1500 mg/L outperforms HPAM-3 at 1200 mg/L, followed by HPAM-1 at 1800 mg/L. Specifically, HPAM-2 at 1500 mg/L achieves the highest incremental recovery of 27.08%, demonstrating the strongest compatibility with the reservoir.

In contrast, the flow diversion results show a different ranking: HPAM-3 at 1200 mg/L exhibits the highest diversion capability, followed by HPAM-2 at 1500 mg/L and HPAM-1 at 1800 mg/L. The maximum diversion ratio of HPAM-3 at 1200 mg/L reaches 58.3%, indicating the most effective flow redistribution toward the low-permeability layer.

By jointly considering both diversion ratio and recovery improvement, HPAM-2 at 1500 mg/L and HPAM-3 at 1200 mg/L were selected for subsequent three-dimensional saturation monitoring well network experiments.

### 3.6. Three Dimensional Saturation Monitoring Well Network Model Oil Displacement Experiment

#### 3.6.1. Dynamic Production Performance

**Figure 13:**
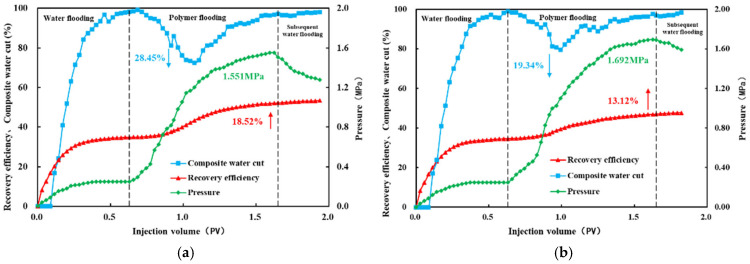
Dynamic production curves from the 3D saturation-monitoring well-pattern displacement tests: (**a**) HPAM-2 at 1500 mg L^−1^; (**b**) HPAM-3 at 1200 mg L^−1^. The arrows indicate the variations in composite water cut and recovery efficiency during the polymer flooding stage.

Based on the results of the preceding experiments, two polymer systems with relatively good compatibility with Class III reservoirs were identified: HPAM-2 at a concentration of 1500 mg/L and HPAM-3 at a concentration of 1200 mg/L. Subsequently, three-dimensional saturation monitoring well network flooding experiments were conducted using these two polymer systems. The corresponding experimental results are shown in Figure 13.

The dynamic production curve for HPAM-2 at 1500 mg/L is shown in Figure 13. During the waterflooding stage, the injection pressure increases slowly and eventually levels off. Initially, only oil is produced with a water cut of zero. The water cut rises rapidly, but the rate of increase slows down as the process continues. During the polymer flooding stage, the injection pressure begins to increase steadily, reaching a maximum pressure of 1.551 MPa. In the polymer injection phase, the water cut decreases gradually before increasing again. The maximum reduction in water cut is 28.45% compared to the waterflooding phase, while the recovery factor improves by 18.52%.

The dynamic production curve for HPAM-3 at 1200 mg/L is shown in Figure 13. The production curve characteristics of both polymer solutions are similar. During the polymer injection phase, the maximum injection pressure for HPAM-3 at 1200 mg/L reaches 1.692 MPa. The maximum reduction in water cut is 19.34% compared to the waterflooding phase, while the recovery factor increases by 13.12%. These results indicate that HPAM-2 at 1500 mg/L exhibits the best compatibility with Class III reservoirs.

#### 3.6.2. Planar Saturation Evolution

Real-time monitoring of the entire displacement process was conducted using an oil saturation monitoring device, and the oil saturation variation patterns for different polymer solutions were plotted [28,29].

**Figure 14:**
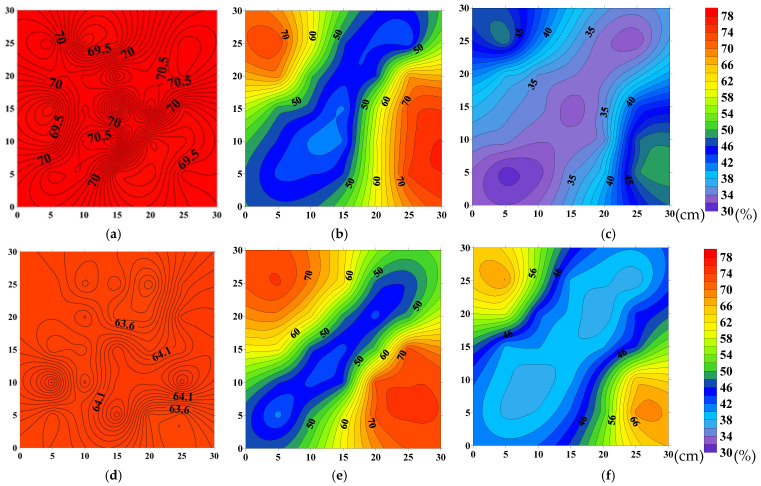
Oil-saturation distributions at successive stages of displacement by HPAM-2 at 1500 mg L^−1^: (**a**) high-permeability layer—initial saturation; (**b**) high-permeability layer—after waterflood; (**c**) high-permeability layer—after polymer flood; (**d**) low-permeability layer—initial saturation; (**e**) low-permeability layer—after waterflood; (**f**) low-permeability layer—after polymer flood.

**Figure 15:**
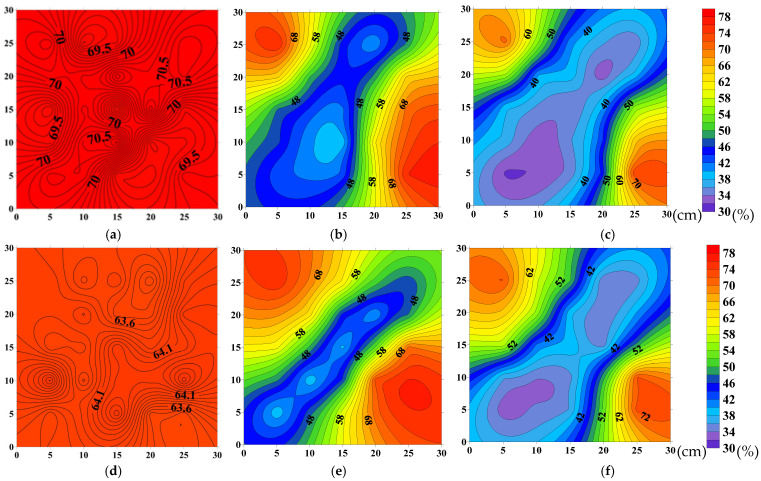
Oil-saturation distributions at successive stages of displacement by HPAM-3 at 1200 mg L^−1^: (**a**) high-permeability layer—initial saturation; (**b**) high-permeability layer—after waterflood; (**c**) high-permeability layer—after polymer flood; (**d**) low-permeability layer—initial saturation; (**e**) low-permeability layer—after waterflood; (**f**) low-permeability layer—after polymer flood.

The oil saturation fields at different displacement stages are shown in Figure 14 and Figure 15. As seen in Figure 14 and Figure 15a–c, during the waterflooding stage, the majority of the high-permeability layer is mobilized, with only small residual oil remaining in the corners. After switching to polymer injection, the oil recovery area further expands under the viscoelasticity of the polymer solution, and additional residual oil in the corners is mobilized.

Figure 14 and Figure 15d–f show that, compared to the oil saturation maps of the high-permeability layer at the same time points, the waterflooding stage primarily mobilizes the high-permeability layer, while the residual oil is relatively enriched in the wings of the low-permeability layer’s main flow lines. After switching to polymer injection, the sweep area of the low-permeability layer is significantly increased due to the improved mobility ratio, and the residual oil in the wings is substantially mobilized.

As shown in Figure 14, in the experiment with HPAM-2 at 1500 mg/L, the recovery factor in the high-permeability layer during the waterflooding stage reached 43.12%, and further increased by 16.24% after switching to polymer injection. In the low-permeability layer, the recovery factor during the waterflooding stage was 28.34%, and after switching to polymer injection, it improved by 18.39%.

As shown in Figure 15, in the HPAM-3 experiment at 1200 mg/L, the recovery factor in the high-permeability layer during the waterflooding stage reached 43.06% and increased by 10.35% after switching to polymer injection. In the low-permeability layer, the recovery factor during the waterflooding stage was 28.18%, and it further increased by 13.19% after switching to polymer injection.

The overall oil displacement efficiency, sweep efficiency, and recovery performance of HPAM-2 at 1500 mg/L in both high- and low-permeability layers are summarized in Table 5. Corresponding performance indicators for HPAM-3 at 1200 mg/L are presented in Table 6.

The saturation distribution images show that the sweep area of the polymer system in the low-permeability layer significantly expanded, and the high residual oil zone was notably reduced. This result is consistent with the sweep efficiency and displacement efficiency data, further validating the enhanced recovery performance.

In summary, for Class III reservoirs in Daqing Oilfield, HPAM-2 at 1500 mg/L outperforms HPAM-3 at 1200 mg/L in terms of enhancing oil recovery.

## 4. Conclusions

This study systematically investigated polymer solution screening and oil recovery performance evaluation for Class III reservoirs in the Daqing Oilfield, and the following conclusions can be drawn:

(1) A comprehensive polymer evaluation method tailored for Class III reservoir conditions was established and applied, based on multiple performance indicators such as viscosity enhancement, stability, shear resistance, injectivity, and profile control capability. The experimental results show that relying on a single performance indicator is insufficient to fully reflect the adaptability of polymer systems in strongly heterogeneous reservoirs. A multi-criteria evaluation approach helps avoid the bias introduced by depending solely on initial viscosity or molecular weight.
(2) Among the 15 polymer solutions tested, the polymer solution with a concentration of 1500 mg/L and a molecular weight of 16 million, and the solution with a concentration of 1200 mg/L and a molecular weight of 19 million, exhibited the best compatibility with Class III reservoir conditions.
(3) In the three-dimensional saturation monitoring well network flooding experiments, the polymer solution with a concentration of 1500 mg/L and a molecular weight of 16 million resulted in a total recovery of 53.38%, with displacement efficiencies of 64.34% and 58.16% for the high- and low-permeability layers, respectively, and sweep efficiencies of 92.26% and 80.35%. The polymer solution with a concentration of 1200 mg/L and a molecular weight of 19 million resulted in a total recovery of 47.71%, with displacement efficiencies of 60.34% and 54.16% for the high- and low-permeability layers, respectively, and sweep efficiencies of 88.52% and 76.38%. The polymer solution with 1500 mg/L concentration and 16 million molecular weight achieved the best improvement in recovery efficiency in Class III reservoirs.
(4) The experimental results further reveal the comprehensive impact of the matching relationship between polymer molecular weight and injection concentration on polymer flooding performance. Excessively high molecular weight or concentration may increase injection resistance, while an inappropriate molecular weight–concentration match makes it difficult to form effective sweep in low-permeability layers. An optimal combination of molecular weight and concentration is one of the key factors in achieving the collaborative mobilization of high- and low-permeability layers in Class III reservoirs.

It should be noted that this study has certain limitations. First, the experimental results are primarily based on core and three-dimensional physical model scales and have not yet been validated with field pilot tests. Second, the effects of polymer adsorption, retention, and the matching relationship between molecular size and pore-throat size have not been quantitatively characterized, and their impact is mainly reflected indirectly through macroscopic oil recovery performance. Additionally, the inversion of oil saturation depends on the resistivity-saturation calibration established under experimental conditions, and its applicability needs further validation under more complex conditions.

Future research will focus on the following directions:

(1) Conducting pilot tests or numerical simulation studies combined with field geological and engineering parameters to verify the field applicability of the experimental conclusions; (2) introducing pore-throat structure characterization and polymer molecular size analysis to further investigate the impact of molecular weight and pore size matching on injectivity and oil recovery performance; (3) combining dynamic injection strategies and multi-scale monitoring methods to improve the comprehensive evaluation system for polymer flooding in strongly heterogeneous reservoirs.

## Figures and Tables

**Table 1:** Reagent and dosage for simulating formation water with a mineralization degree of 6778 mg/L.

Reagent Concentration	NaHCO_3_	NaCl	KCl	MgSO_4_	Na_2_SO_4_	CaCl_2_
**Salinity (** **mg L^−1^** **)**	2829	3489	20	262	114	64

**Table 2:** Shear resistance experiment—one-dimensional long core parameters.

Group Number	Permeability (×10^−3^ μm^2^)	Size (mm) Length × Width × Height	Porosity (%)
1	100	300 × 45 × 45	25.84
2	100		26.17
3	100		26.34

**Table 3:** Dual tube parallel core parameters.

Group Number	Permeability (×10^−3^ μm^2^)	Size (mm) Length × Width × Height	Porosity (%)	Permeability Ratio	Oil Saturation (%)
1	100	300 × 45 × 45	26.19	2	73.72
	200		29.79		76.24
2	100	300 × 45 × 45	25.51	2	77.44
	200		28.48		78.94
3	100	300 × 45 × 45	26.35	2	75.68
	200		28.92		76.51

**Table 4:** Parameters of 3D saturation monitoring well network model.

Model 1	Permeability (×10^−3^ μm^2^)	Size (mm) Length × Width × Height	Porosity (%)	Oil Saturation (%)
low permeability layer	102	300 × 300 × 22.5	26.07	66.86
High permeability layer	205	300 × 300 × 22.5		
**Model 2**	**Permeability (×10^−3^ μm^2^)**	**Size (mm)** **Length × Width × Height**	**Porosity (%)**	**Oil Saturation (%)**
low permeability layer	101	300 × 300 × 22.5	25.97	67.43
High permeability layer	203	300 × 300 × 22.5		

**Table 5:** Petrophysical and displacement parameters of the high- and low-permeability layers before and after HPAM-2 at 1500 mg L^−1^.

	Oil Displacement Efficiency (%)	Sweep Efficiency (%)	Recovery Efficiency (%)	Total Recovery Rate (%)
High permeability layer	64.34	92.26	59.36	53.38
Low permeability layer	58.16	80.35	46.73	

**Table 6:** Petrophysical and displacement parameters of the high- and low-permeability layers before and after HPAM-3 at 1200 mg L^−1^.

	Oil Displacement Efficiency (%)	Sweep Efficiency (%)	Recovery Efficiency (%)	Total Recovery Rate (%)
High permeability layer	60.34	88.52	53.41	47.71
Low permeability layer	54.16	76.38	41.37	

## Data Availability

The raw data supporting the conclusions of this article will be made available by the authors on request.

## Abbreviations

The following abbreviations are used in this manuscript:

HPAM	Hydrolyzed polyacrylamide
HPAM-1	Hydrolyzed Polyacrylamide with a molecular weight of 12 million
HPAM-2	Hydrolyzed Polyacrylamide with a molecular weight of 16 million
HPAM-3	Hydrolyzed Polyacrylamide with a molecular weight of 19 million
PV	Pore volume
3D	Three-dimensional
EOR	Enhanced oil recovery
ISCO	ISCO syringe pump
MPa	Megapascal
mPa·s	Millipascal second
°C	Degree Celsius
E_D_	Oil displacement efficiency
E_V_	Sweep efficiency
